# Mechanical Analysis for Active Movement of Upper Limb Rehabilitation Robots to Alleviate Shoulder Pain in Patients with Stroke Hemiplegia and Frozen Shoulder

**DOI:** 10.3390/s25216644

**Published:** 2025-10-30

**Authors:** Seok Jin Bang, Jung-Soo Lee, Dong Hyeon Song, Seung Yeob Ryu, Kwang Gi Kim

**Affiliations:** 1 KMAIN Co., Ltd., Seongnam 13517, Republic of Korea; sj97014@gmail.com; 2 Medical Devices R&D Center, Gachon University Gil Medical Center, Incheon 21565, Republic of Korea; 3 Department of Biomedical Engineering, Gachon University College of Medicine, Incheon 21565, Republic of Korea; 4 Biohealth & Medical Engineering Major, Gachon University, Seongnam 13120, Republic of Korea; 5 Gachon Biomedical & Convergence Institute, Gachon University Gil Medical Center, Incheon 21565, Republic of Korea; 6 Department of Health Sciences and Technology, Gachon Advanced Institute for Health & Sciences and Technology (GAIHST), Seongnam 13120, Republic of Korea

**Keywords:** robot, rehabilitation, mechanics, BioHealth, physical therapy

## Abstract

Shoulder disorders, including frozen shoulder resulting from stroke-induced hemiplegia, significantly reduce a patient’s ability to perform activities of daily living, thereby necessitating repeated rehabilitation. Consequently, extensive research has been conducted on rehabilitation robots to assist in upper-limb motor recovery. The shoulder moves according to the scapulohumeral rhythm. Considering the biomechanical characteristics of the shoulder joint, the rehabilitation robot was designed to replicate a similar kinematic environment using actuators and linkages that emulate the structures of the upper arm, shoulder, and clavicle. To ensure precise operation, the kinematic accuracy of the robot was pre-evaluated. Kinematic analyses were conducted using MATLAB, and the results were compared with coordinate data from the mechanical design to evaluate positional accuracy. In addition, the convergence and accuracy of joint-angle estimation for target positions were analyzed. The forward kinematic analysis revealed that the average positional error between the measured and target coordinates ranged from 0.5% to 2.8%, with the Base Motor–Back Motor segment exhibiting the highest error (2.8%). The inverse kinematic analysis demonstrated stable convergence to the target positions through iterative computations using the Gauss–Newton method, confirming that the actual motion could be accurately reproduced within the designed range of motion.

## 1. Introduction

The prevalence of shoulder-related disorders, such as stroke and frozen shoulder, is steadily increasing in Korea [1], while the age of onset has gradually decreased [2]. Stroke causes upper limb paralysis and impaired motor function, significantly limiting an individual’s ability to perform daily activities [3]. Frozen shoulder restricts joint movement and causes pain, requiring long-term rehabilitation [4]. Impaired shoulder joint function [5,6] is common to these conditions, necessitating systematic rehabilitation therapy. However, owing to the shortage of rehabilitation personnel and time constraints, providing personalized therapy that reflects each patient’s condition remains challenging [7].

To overcome these limitations, rehabilitation robots have been developed to deliver repetitive and quantifiable movements [8,9]. Several upper-limb systems, including MIT Manus (InMotion), DIEGO (Tyromotion), ReoGo-J (Motorika), and Armeo^®^Power (Hocoma), have demonstrated clinical efficacy [10,11,12,13]. However, these devices present trade-offs between cost, complexity, and motion capability: simpler systems such as MIT Manus offer limited planar motion, whereas high-performance exoskeletons like DIEGO and Armeo^®^Power enable multi-joint movements but are expensive, require large installation spaces, and demand skilled operation [10,11,12,13].

IntelliArm is an exoskeleton-type rehabilitation robot that integrates diagnostic, therapeutic, and evaluation functions through multiple sensors and actuators [14]. Although such advanced systems achieve high precision, their cost and structural complexity hinder widespread clinical adoption. Furthermore, even wearable robots with improved safety and usability still face challenges in precise trajectory control and in reflecting voluntary patient intent [15]. Balancing biomechanical accuracy, responsiveness, and cost-effectiveness remains an unresolved issue [16].

To address this gap, our research team developed an affordable upper-limb rehabilitation robot that reflects the patient’s anatomy and shoulder biomechanics. Usability evaluation results showed positive feedback from both patients and clinicians regarding stability and assistance [17]. The current system supports passive and stretching modes that operate along predefined trajectories; however, to realize an active-assisted mode that responds to voluntary motion, precise kinematic modeling and mechanical accuracy must be ensured [18].

The shoulder joint has the highest degrees of freedom (DOF) among human joints, with the scapula, humerus, and clavicle interacting to dynamically change the center of motion [19]. Therefore, accurate forward and inverse kinematic modeling—capturing the position and rotational relationships of each joint—is essential for reproducing natural biomechanics [20]. In particular, precise estimation of the end-effector position based on link lengths, motor angles, and coordinate transformations is a key prerequisite for implementing active-assisted control [21].

This study aimed to develop an upper-limb rehabilitation robot that reproduces natural shoulder motion based on the scapulohumeral rhythm, reflecting the biomechanics of patients and establishing a foundation for implementing an active-assisted control mode. To achieve this, a 4-motor-based prototype capable of passive and stretching modes was designed to be lightweight and cost-efficient while maintaining functional performance. Forward kinematic analysis using Euler angles was conducted to verify positional accuracy by comparing the MATLAB (R2023b; MathWorks, Natick, MA, USA) analysis results with SolidWorks (2018; Dassault Systèmes, Vélizy-Villacoublay, France) simulation data, and the inverse kinematics was solved using the Gauss–Newton method to validate the accuracy of angle estimation.

Unlike conventional upper-limb rehabilitation robots that mainly focus on planar or shoulder–elbow movements, the proposed system uniquely incorporates scapulohumeral rhythm to reproduce physiologically accurate shoulder motion and minimize compensatory movement. The developed prototype serves as a foundational platform for realizing an affordable, clinically accessible, and actively assisted rehabilitation system.

The remainder of this paper is organized as follows: Section 2 describes the methodology, including the explanation of the scapulohumeral rhythm applied to the robot design, the usability evaluation setup, and the kinematic computation process. Section 3 presents the results of the forward and inverse kinematic analyses and compares the calculated coordinates with those obtained from SolidWorks simulations to verify model accuracy. Section 4 discusses the findings, highlighting the implications and limitations of the proposed approach, and Section 5 concludes the paper with perspectives on future work toward active-assisted control implementation.

## 2. Method

### 2.1. Scapulohumeral Rhythm

**Figure 1:**
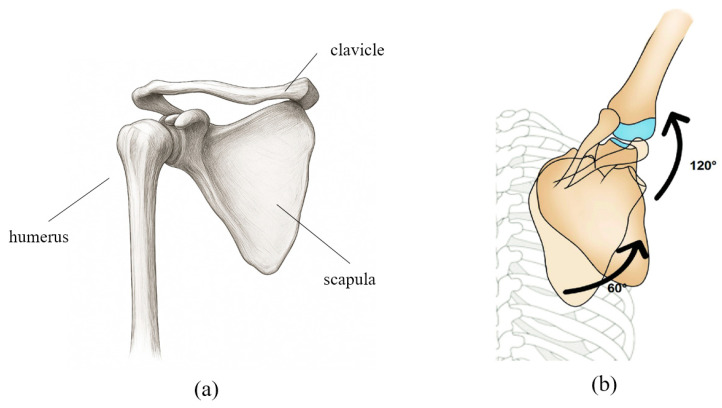
(**a**) Schematic representation of the anatomical structure of the shoulder bone. (**b**) The movement of each joint and bone according to the scapulohumeral rhythm is shown in degrees, with the shoulder bone and upper arm bone moving at a ratio of approximately 1:2.

For effective shoulder joint rehabilitation, it is essential to understand and replicate the biomechanical movements of the shoulder complex. Figure 1a illustrates the anatomical structure of the shoulder girdle, which enables complex, coordinated movements among multiple joints. The shoulder motion involves rotation of the humerus accompanied by simultaneous movements of the scapula and clavicle, resulting in a shifting center of rotation. This coordinated movement is described by the scapulohumeral rhythm. As shown in Figure 1b, during 180° of arm abduction, the humerus and scapula move in a ratio of approximately 2:1 (120° and 60°, respectively), with simultaneous upward rotation of the scapula [22].

This biomechanical relationship must be considered when defining the range of motion (ROM) for the robot. Accordingly, an ROM model was established for each representative posture of the patient’s shoulder movement to be applied to the robotic system. In addition, motion data for each shoulder movement were collected from healthy individuals to provide reference trajectories, which were then converted into text-based datasets and implemented in the robot’s passive and stretching modes to enable natural and patient-specific movement patterns. However, although the robot could reproduce fixed postures based on these parameters, additional mechanical verification was required to allow the patient to move the robot freely. Therefore, a mechanical analysis of the designed robot was conducted.

**Figure 2:**
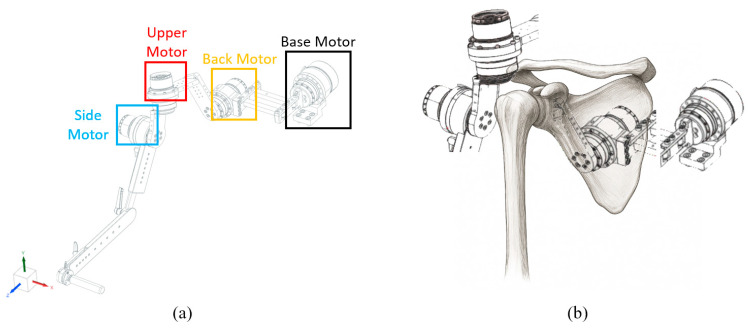
(**a**) Robot design. The base motor corresponds to the center axis of the body, and each motor corresponds to the Back Motor corresponding to the scapula, the upper motor corresponding to the upper arm bone, and the side motor corresponding to the side of the upper arm bone. (**b**) Different motors corresponding to the shoulder bone position.

The robot designed according to this configuration is shown in Figure 2. To reproduce the scapulohumeral rhythm, the motion of each anatomical segment was actuated by four motors. As illustrated in Figure 2, the Base Motor, Back Motor, Upper Motor, and Side Motor were configured to enable coordinated replication of complex shoulder movements.

### 2.2. Usability Evaluation

We conducted a usability evaluation of the prototype robot currently under development to assess its suitability in comparison with a version that did not include the active-assisted mode. The evaluation was performed in the passive and stretching modes. It should be noted that this usability study was conducted prior to the present research using the same robotic platform, serving as a preliminary assessment to verify its functional applicability and user safety before implementing the active-assisted mode.

#### 2.2.1. Selection of Subjects

Fifteen patients with upper-limb disabilities participated in the study, including seven diagnosed with shoulder dislocation and eight with hemiplegia resulting from stroke. In patients with hemiplegia resulting from stroke, evaluations were conducted based on the Modified Ashworth Scale (MAS), targeting individuals classified as Grade 2 or below among Grades 1–5. Patients with Grade 3 or higher were excluded, as wearing the medical device could pose a risk of injury or aggravation of existing conditions. Accordingly, the inclusion criteria were limited to patients capable of voluntary movement and able to perceive external stimuli. All procedures were approved by the Institutional Review Board (IRB), and all participants provided written informed consent before participation.

Before using the equipment, participants received an instruction manual describing the structure of the device, operation procedures, study objectives, expected benefits, and potential risks. Training sessions were conducted to ensure that each participant could operate the system safely and effectively.

Patients with severe hemiplegia due to stroke, for whom it was difficult to apply the rehabilitation treatment scenario, were excluded from the clinical trial. Additionally, patients were selected based on the stage of adhesive capsulitis—freezing, frozen, or thawing—so that appropriate rehabilitation treatment protocols could be prescribed according to their individual condition.

#### 2.2.2. Selection of Subjects and Ethical Considerations

Fifteen patients with upper-limb disabilities (seven with shoulder dislocation and eight with hemiplegia) participated in the study, and all research procedures were conducted after approval by the Institutional Review Board (IRB).

Patients with stroke were limited to those in the chronic phase, defined as more than six months after onset, who were fully conscious and able to follow instructions. Patients with frozen shoulder were included if adhesive capsulitis was confirmed through imaging examinations and clinical diagnosis. Conversely, individuals with acute inflammation, a history of shoulder trauma or surgery within the previous six months, severe pain (NRS ≥ 7), or mental or cognitive impairments that prevented them from following instructions were excluded.

#### 2.2.3. Experimental Equipment and Motion Protocol

The shoulder rehabilitation robot used in this study was designed to perform six upper-limb movements: adduction, abduction, extension, flexion, internal rotation, and external rotation.

Patients with stroke were limited to those in the chronic phase, defined as more than six months after onset, who were fully conscious and able to follow instructions. Patients with frozen shoulder were included if adhesive capsulitis was confirmed through imaging examinations and clinical diagnosis. Conversely, individuals with acute inflammation, a history of shoulder trauma or surgery within the previous six months, severe pain (NRS ≥ 7), or mental or cognitive impairments that prevented them from following instructions were excluded. According to consultation with a rehabilitation medicine specialist, the average patient performed three sets of ten repetitions per exercise, which served as the basis for the training protocol. However, because symptoms and pain levels varied among individuals, the number of repetitions and exercise modes were adjusted under the supervision of a physical therapist. The program was applied to patients with hemiplegia caused by stroke and those diagnosed with adhesive capsulitis.

All participants underwent a preliminary assessment to quantitatively evaluate pain levels and pathological symptoms, including restricted range of motion (ROM), increased muscle tone, and muscle weakness. Based on these results, a rehabilitation mode suitable for each patient’s condition was prescribed. Specifically, the Passive Mode was applied to patients with severe impairment who had difficulty performing active movements, while the Stretching Mode was used concurrently to enhance joint flexibility and alleviate muscle tension.

Each mode was continuously adjusted by monitoring the patient’s pain response and pathological muscle tone level. Pre- and post-assessments focused on the Visual Analogue Scale (VAS) for pain and the range of motion (ROM). Through this process, individualized exercise prescriptions were developed according to changes in symptoms, thereby verifying the clinical effectiveness of the robot-assisted rehabilitation program.

#### 2.2.4. Data Collection and Processing

Range of Motion (ROM) data were collected through preliminary task performance, during which more than 30 datasets were obtained. These data were then used for control group comparison in the present clinical study. The study was conducted through multidisciplinary collaboration involving physical therapists, rehabilitation medicine specialists, researchers, stroke patients with hemiplegia, and patients diagnosed with adhesive capsulitis, each contributing to data acquisition and validation according to their respective roles.

During data collection, both initial and post-rehabilitation ROM values were manually assessed and recorded in case report forms (CRFs). The improvement in ROM was evaluated by comparing the Initial Range of Motion (Initial ROM), Target Range of Motion (Target ROM), and Post-Rehabilitation Range of Motion (Post-ROM), as documented in the CRFs.

In addition, prior discussions were held with patients and their caregivers to verify symptoms and prevent potential safety issues, taking into account that assessment values and Visual Analogue Scale (VAS) scores for pain may differ depending on whether the rehabilitation or physical therapy period exceeded six months.

### 2.3. Kinematic Analysis Verification for Active Assisted Mode

#### 2.3.1. Robot Coordinate System Analysis

In the previously implemented passive and stretching modes, the robot operated along fixed trajectories; therefore, smooth operation could be confirmed without performing kinematic analysis. In contrast, the active-assisted mode requires kinematic analysis that accounts for each motor and link to accurately reproduce the motion of the robot’s end effector.

**Figure 3:**
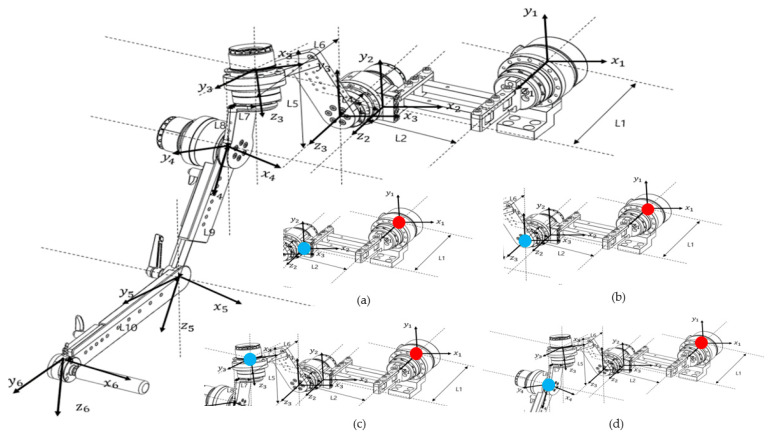
Diagram showing the coordinate axes for each motor based on Euler angles for kinematic analysis, as indicated on the design drawing. The diagram at the bottom right shows the calculation of the end effector position for each part from each reference point. (**a**) Base Motor–Link2, (**b**) Base Motor–Back Motor, (**c**) Base Motor–Upper Motor, and (**d**) Base Motor–Side Motor (red dots indicate reference points and blue dots indicate end effectors).

Before conducting the kinematic analysis, the coordinate frames of the robot were defined. The coordinate system of the end effector was sequentially established with reference to the base motor, and the final coordinate configuration is illustrated in Figure 3.

There are two general approaches to geometric modeling of robot manipulators: one uses Denavit–Hartenberg (D–H) parameters, and the other employs Euler angles. The coordinate frame of each link is defined based on these parameters and used to calculate the overall transformation that determines the position of the robot’s end effector. This process is typically performed using homogeneous transformation matrices.

In this study, because the robot’s links were connected at angular offsets rather than in a simple serial configuration, forward kinematics were analyzed using Euler angles to describe the manipulator’s orientation. Three factors were considered in this analysis: (1) the rotation matrix of each coordinate frame, (2) the rotation of the coordinate axes according to motor rotation, and (3) the position vector of each link. By successively multiplying the homogeneous transformation matrices that incorporate these parameters, the final position and orientation of the end effector were obtained.

#### 2.3.2. Regular Geometry Calculation

**Figure 4:**
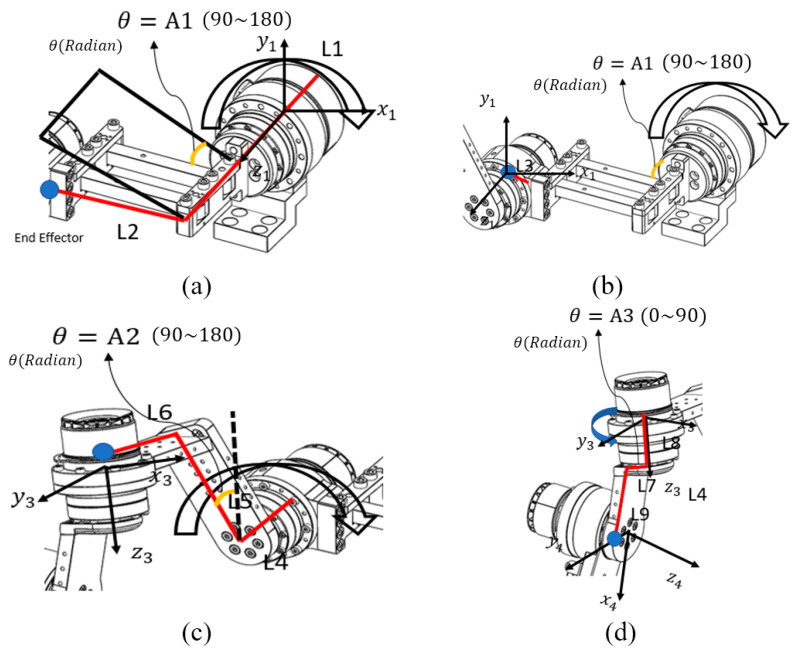
Coordinate axis analysis for each motor: (**a**) Base Motor–Link 2, (**b**) Link 2–Back Motor, (**c**) Back Motor–Upper Motor, and (**d**) Upper Motor–Side Motor. Each part represents the divided end-effector calculation for the corresponding motor segment. The red lines indicate the links used in the coordinate calculation, while the blue dots represent the end-effector positions of each segment.

The analysis of the robot’s end effector was performed starting from the base motor. As described in Figure 4, the calculation process was divided into four parts corresponding to each joint, and sequential calculations were performed to verify the accuracy of the derived equations. The rectangular link connected to the base motor was designated as the first end-effector, and its kinematic behavior was analyzed as the initial step of the process.

Abbreviations used in the equations were defined according to the characteristics they represent. “rot” refers to *rotation*, indicating the directional changes in the x-, y-, and z-axes with respect to the previous coordinate frame. “rotmat” denotes the *rotation matrix*, which contains information about the motor’s angular orientation within the coordinate system. “div” represents *divergence*, describing the position vector of a link resulting from the motor’s rotation. The homogeneous transformation matrix is referred to as the Homogeneous Matrix, and the combined transformation matrix integrating all these elements is denoted as “Homgen”. In order to distinguish the matrices corresponding to each end-effector, subscripts were added to the variable names to indicate which end-effector’s values were being calculated.

The first step of the end-effector analysis involved the Base Motor–Link2 segment. As shown in Figure 4a, each link was defined for analysis, and the motor rotation angle for this segment was designated as *A*_1_. Because the coordinate frame of the Base Motor–Link2 section represents the reference motor coordinate system, it can be expressed as an identity matrix, as shown below:

(1) $$rot_{01}=\left[\begin{array}{ccc} 1 & 0 & 0\\ 0 & 1 & 0\\ 0 & 0 & 1 \end{array}\right]$$

Base Motor—In the case of the rotation matrix, considering the rotation of the motor in Link2, because the motor axis rotates around the z-axis, the Euler angle representing the z-axis rotation must be multiplied in Equation (1). However, because this is a reference coordinate system, the coordinate axes do not change when the motor rotates; therefore, they can be represented by an identity matrix as follows:

(2) $${rotmat}_{01}=\left[\begin{array}{ccc} 1 & 0 & 0\\ 0 & 1 & 0\\ 0 & 0 & 1 \end{array}\right]$$

For the Base Motor–Link2 segment, the position vector of the end effector can be expressed as shown in Equation (3), because the position of the end effector varies with the rotation angle *A*_1_, as illustrated in Figure 4.

(3) $${div}_{01}=\left[\begin{array}{c} L2\cos\left(A1\times rad\right)\\ L2\sin\left(A1\times rad\right)\\ L1 \end{array}\right]\;(rad=\frac{pi}{180})$$

In summary, the transformation matrix from Base Motor to Link2 is given by Equation (4) as follows:

(4) $$Homgen_{01}=\left[\begin{array}{cccc} 1 & 0 & 0 & L2\cos\left(A1\times rad\right)\\ 0 & 1 & 0 & L2\sin\left(A1\times rad\right)\\ 0 & 0 & 1 & L1\\ 0 & 0 & 0 & 1 \end{array}\right]$$

Subsequently, the midpoint of the second motor from the Base Motor was defined as the second end effector, and the analysis was continued. As shown in Figure 4, the link length and motor rotation angle were defined, and the transformation matrix for calculating the kinematic relationship from the Base Motor to the Back Motor was derived as follows.

The second end-effector analysis corresponded to the Base Motor–Back Motor segment. For this configuration, the distance between the end of Link 2 and the Back Motor was defined as Link 3. The coordinate frame of the Base Motor–Back Motor segment was identical to the reference motor coordinate system; therefore, the transformation could be represented by an identity matrix.

(5) $${rot}_{12}=\left[\begin{array}{ccc} 1 & 0 & 0\\ 0 & 1 & 0\\ 0 & 0 & 1 \end{array}\right]$$

The rotation matrix that accounts for motor rotation in the Base Motor–Back Motor segment is expressed in Equation (6). In this case, the rotation of the Base Motor influences the orientation of the end-effector coordinate frame; therefore, the motor rotation must be incorporated into the rotation matrix.

(6) $${rotmat}_{12}=\left[\begin{array}{ccc} cos(A1-180\times rad) & -sin(A1-180\times rad) & 0\\ sin(A1-180\times rad) & cos(A1-180\times rad) & 0\\ 0 & 0 & 1 \end{array}\right]\times \left[\begin{array}{ccc} 1 & 0 & 0\\ 0 & 1 & 0\\ 0 & 0 & 1 \end{array}\right]$$

The position vector of the end effector for the Base Motor–Back Motor segment is expressed as in Equation (7), where only *L*_3_ is applied.

(7) $${div}_{12}=\left[\begin{array}{c} L3\cos\left(A1\times rad\right)\\ L3\sin\left(A1\times rad\right)\\ 0 \end{array}\right]$$

In summary, the transformation matrix from the Base Motor to the Back Motor is expressed as shown in Equation (8).

(8) $$Homgen_{12}=\left[\begin{array}{cccc} & & & L3\cos\left(A1\times rad\right)\\ & {rotmat}_{12} & & L3\sin\left(A1\times rad\right)\\ & & & 0\\ 0 & 0 & 0 & 1 \end{array}\right]$$

By multiplying Equations (4) and (8), the rotation matrix and position vector of the end effector from the Base Motor to the Back Motor were obtained. The variations in the end-effector position resulting from the link lengths and motor angles were incorporated into the equation. Using this approach, the coordinate values of the robot’s end effector—driven by the base and side motors—were calculated based on the homogeneous transformation matrices obtained for each configuration. Forward geometric analysis was then performed to implement the active-assisted mode.

(9) $$Homgen_{02}=Homgen_{01}\times {Homgen}_{12}$$

#### 2.3.3. Verification of Regular Kinematics Through Inverse Kinematics

**Figure 5:**
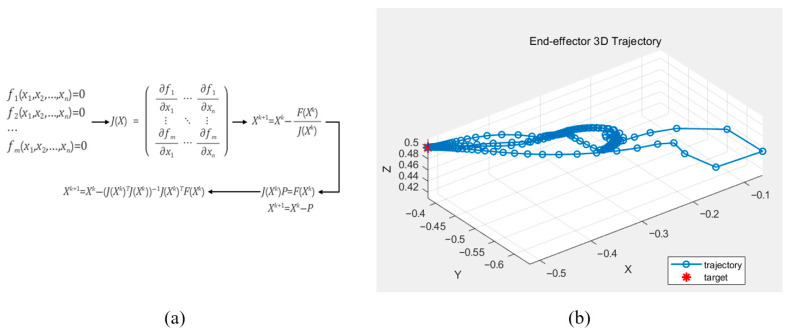
The Gauss–Newton method for calculating the rigid body kinematics (**a**) and the calculated end-effector position displacement based on the computed rigid body kinematics (**b**).

To verify the forward kinematics, inverse kinematic calculations were performed using the Gauss–Newton method, which is commonly employed to approximate solutions to systems of nonlinear equations. The Gauss–Newton algorithm estimates model parameters by solving a nonlinear least-squares problem, as illustrated in Figure 5. Using this method, the joint angles required for the robot’s end effector to reach a desired position within a specified number of iterations were determined. The Gauss–Newton method was implemented in MATLAB, and the computed results confirmed that the derived equations accurately produced the joint angles corresponding to the target positions.

The above equation represents the mathematical process of the Gauss–Newton method used to validate the derived kinematic equations.

## 3. Result

To verify the kinematic accuracy of the upper-limb rehabilitation robot, the end-effector positions of each motor segment were compared between the theoretical analysis results obtained using MATLAB and the measured position values derived from SolidWorks simulations. The theoretical positions were calculated through Euler-angle-based forward kinematic analysis, and the measured values were extracted from the SolidWorks simulation under identical angular conditions. This comparison enabled quantitative evaluation of the positional errors for each segment, as summarized in Table 1.

Table 1 presents the positional errors for each segment and compares the maximum error rates between the SolidWorks simulation and MATLAB analysis results. The maximum error in this table refers to the largest deviation among the x-, y-, and z-axis positional differences between the two models. The maximum and minimum errors were 2.8% and 0.5%, respectively, confirming that the forward kinematic analysis was performed accurately. The higher error observed in the Base Motor–Back Motor segment is attributed to the end effector being defined at a suboptimal position relative to the coordinate system and link geometry. However, even small deviations in joint positioning may lead to unexpected movement trajectories in a shoulder-driven robotic system, potentially posing a risk of unintended strain or injury to the patient. Therefore, minimizing these errors is critical for ensuring both the accuracy and safety of rehabilitation exercises.

**Figure 6:**
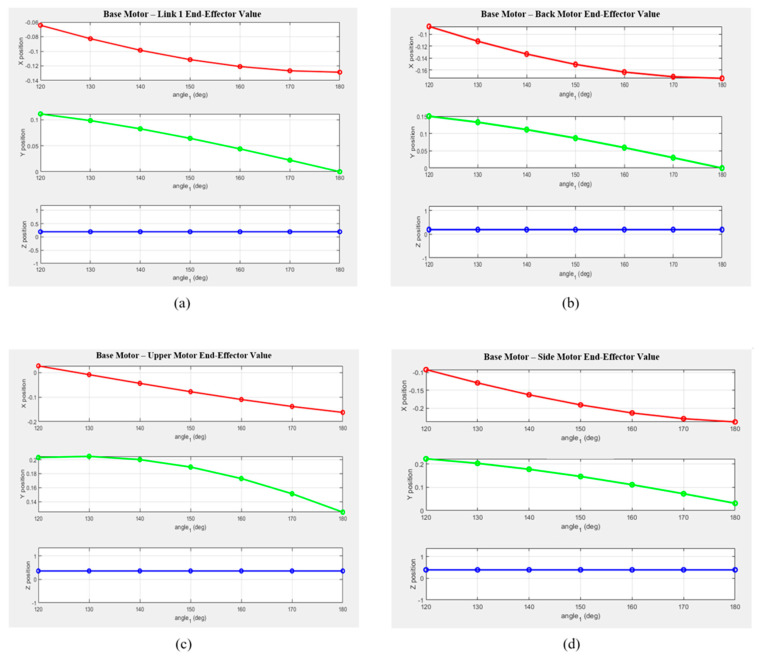
Variations in the end-effector position along the x-, y-, and z-axes for each drive motor configuration when the base motor angle changes from 10° to 60°: (**a**) from the Base Motor to Link 1; (**b**) from the Base Motor to the Back Motor; (**c**) from the Base Motor to the Upper Motor; and (**d**) from the Base Motor to the Side Motor.

Figure 6 presents the MATLAB-generated graphs showing the variations in the X, Y, and Z coordinates for each segment as a function of the rotation angle. Based on the theoretical values, it can be visually observed that the position of the end effector changes according to the motor rotation angle. The graphs illustrate the positional changes along each axis for every segment, revealing that variations along the Z-axis were minimal.

- The X-coordinate exhibited a decreasing trend with a negative slope across most segments, with notable deviations observed in Links 2 and 3.
- The Y-coordinate showed a nearly linear decrease as the rotation angle increased, suggesting a close relationship with the motion of the rotational center.
- The Z-coordinate exhibited only minor changes across all segments, indicating that the robot structure was primarily designed for planar motion.

Consequently, the kinematic analysis of the robot developed in this study was quantitatively validated. It was confirmed that specific formulations are required for points where the links and coordinate axes are parallel and share the same reference frame. In sections involving multi-axis rotation—such as between the Back Motor and Upper Motor, and between the Upper Motor and Side Motor—the calculated results for coordinate rotation were found to be accurate, consistent with the results presented in Table 2. Future research will focus on achieving higher precision by applying coordinate-frame calibration, optimizing rotation sequences, and introducing measurement-based correction algorithms in the multi-axis rotation segments to further minimize errors.

## 4. Discussion

This study aimed to develop and validate a biomechanically inspired upper-limb rehabilitation robot that reproduces natural shoulder motion based on the scapulohumeral rhythm, thereby laying the groundwork for implementing an active-assisted control mode. By designing and mechanically analyzing an upper-limb rehabilitation robot for patients with stroke and frozen shoulder, this study established a foundation for implementing an active-assisted mode based on admittance control. Among previously developed rehabilitation robots, the MIT Manus (InMotion) performs only simple planar movements using a two-degree-of-freedom (DOF) structure, whereas high-performance systems such as DIEGO and Armeo^®^Power are equipped with precise sensors and safety mechanisms but are costly due to their complex configurations. Consequently, their high cost and large installation space requirements hinder widespread clinical adoption.

To address these limitations, an affordable upper-limb rehabilitation robot was developed in this study, reflecting the patient’s body structure and shoulder biomechanics. Its usability was verified through user testing. The robot was designed to be compact for use in limited spaces while being capable of replicating complex shoulder movements. However, the current functionality is limited to position control within a predefined range of motion (ROM). Therefore, this study analyzed the coordinate systems of the main links and motor configurations of the robot—designed to replicate the anatomical structure of the shoulder joint—and performed inverse kinematic analysis using forward kinematics and homogeneous transformation matrices.

During the analysis, the positions of each end effector were mathematically derived based on motor rotation angles, link lengths, and coordinate transformations. The calculated position values were then compared with those obtained from the SolidWorks simulation to evaluate accuracy. The average positional error between theoretical and simulated values ranged from 0.5% to 1% across all segments from the Base Motor to the Side Motor, indicating good agreement. In the Upper Motor–Side Motor section, the largest error among the four segments was observed due to minor interference between rotation axes and the simplified structure of the straight link when analyzed using the same coordinate frame. In contrast, the Back Motor section exhibited a relatively small error (≤0.5%), which can be attributed to mathematical compensation and accurate angle verification despite its multi-axis configuration. These findings highlight subtle differences between the physical links and the theoretical coordinate system.

Overall, the results validate the kinematic analysis and emphasize the importance of carefully defining coordinate systems and end-effector positions for each segment during robot design. To implement an active-assisted mode that responds to the patient’s voluntary motion, the end effector must accurately reach the target position; accumulated errors during this process can lead to unintended movements, directly affecting patient safety. This underscores that precise kinematic modeling is not only essential for design validation but also critical for ensuring clinical safety.

Furthermore, this precise kinematic modeling is particularly critical for implementing advanced control strategies such as admittance or impedance control, which require the robot to respond stably to external forces and user-generated interactions. In such control modes, any inaccuracy in the geometric model or coordinate transformation may lead to unintended force responses or unstable motion behavior, potentially compromising both safety and therapeutic effectiveness. Therefore, the verified accuracy of the present kinematic analysis provides a crucial foundation for future development of active-assisted rehabilitation algorithms based on admittance or impedance control.

This underscores that precise kinematic modeling is not only essential for design validation but also critical for ensuring clinical safety.

Furthermore, the proposed design improves the robot’s wearability and can provide enhanced assistance for patients with asymmetric muscle distribution in the upper limbs. However, for real-world clinical applications, both mechanical precision and patient-specific adaptability are crucial. The current robot features a fixed link length based on average body dimensions, limiting its ability to accommodate individual anatomical differences. If these structural improvements are implemented, the system could actively adapt to the diverse body sizes of individual users, thereby reducing position estimation errors and enabling more accurate active-assisted control. Such adaptability would also minimize the potential risk of secondary injuries, particularly for patients who may be sensitive to unintended joint stresses or excessive motion. Future work should focus on improving the link length adjustment mechanism by adopting a modular design that enables easy manual adjustment, or by implementing a user-customized fitting system optimized for individual shoulder geometries. In addition, future research will focus on achieving higher precision by applying coordinate-frame calibration, optimizing rotation sequences, and introducing measurement-based correction algorithms in the multi-axis rotation segments to further minimize errors. In particular, introducing physical adjustment mechanisms such as detachable auxiliary links or micro-adjusters is technically feasible and expected to yield significant benefits when integrated with future adaptive control algorithms.

## 5. Conclusions

In conclusion, this study demonstrated that precise kinematic analysis of upper-limb rehabilitation robots is essential for implementing admittance-based active-assist control. The validity of the proposed kinematic analysis was verified by comparing the analytical results with actual measurement data. Future studies will focus on minimizing analysis errors in specific segments to further enhance the accuracy and safety of rehabilitation robots. The findings of this study can serve as a technical reference for the future development of patient-specific rehabilitation platforms and are expected to contribute to the broader clinical adoption of robot-assisted rehabilitation therapy.

## Figures and Tables

**Table 1:** Maximum error rate and maximum error for each interval.

Section	Maximum Error Rate (%)	Maximum Error (cm)	Main Causes of Errors
Base Motor–Link1	0.6%	0.08	Numerical differences between simulation and MATLAB
Base Motor–Back Motor	2.8%	0.49	Error in changing formulas on the same line
Base Motor–Upper Motor	0.5%	0.08	Multi-axis rotation
Base Motor–Side Motor	0.5%	0.22	Multi-axis rotation

**Table 2:** Summary of errors for each section. The table has been divided into ranges of 10 to 30° and 30 to 60°.

Base Motor -> link2	MATLAB	SolidWorks	Result	MATLAB	SolidWorks	Result	MATLAB	SolidWorks	Result
Degree	10	10		20	20		30	30	
x	−12.68	−12.67	0.01	−12.1	−12.09	0.01	−11.15	−11.17	0.02
y	2.23	2.17	0.06	4.33	4.4	0.07	6.43	6.35	0.08
z	19.55	19.55	0	19.56	19.55	−0.01	19.56	19.55	−0.01
Base Motor -> Back Motor	MATLAB	SolidWorks	Result	MATLAB	SolidWorks	Result	MATLAB	SolidWorks	Result
Degree	10	10		20	20		30	30	
x	−17.11	−16.62	0.49	−16.32	−15.87	0.45	−15.04	−14.64	0.4
y	3.02	2.86	0.16	5.94	5.69	0.25	8.68	8.35	0.33
z	19.55	19.44	0.11	19.55	19.44	0.11	19.55	19.45	0.1
Base Motor -> Upper Motor	MATLAB	SolidWorks	Result	MATLAB	SolidWorks	Result	MATLAB	SolidWorks	Result
Degree	10	10		20	20		30	30	
x	−13.81	−13.89	0.08	−10.97	−11.05	0.08	−7.8	−7.88	0.08
y	15.13	15.12	0.01	17.3	17.28	0.02	18.94	18.92	0.02
z	36.23	36.24	0.01	36.23	36.25	0.02	36.23	36.27	0.04
Base Motor -> Side Motor	MATLAB	SolidWorks	Result	MATLAB	SolidWorks	Result	MATLAB	SolidWorks	Result
Degree	10	10		20	20		30	30	
x	−22.9	−22.84	0.07	−21.3	21.24	0.06	−19.06	−19	0.06
y	7.22	7.19	0.03	11.09	11.02	0.07	14.62	14.52	0.1
z	39.36	39.14	0.22	39.36	39.15	0.21	39.36	39.16	0.2
Base Motor -> link2	MATLAB	SolidWorks	Result	MATLAB	SolidWorks	Result	MATLAB	SolidWorks	Result
Degree	40	40		50	50		60	60	
x	−9.89	−9.85	0.03	−8.32	−8.27	0.05	−6.49	−6.43	0.06
y	8.18	8.27	0.09	9.76	9.85	0.1	11.05	11.15	0.1
z	19.57	19.55	−0.02	19.58	19.55	−0.03	19.58	19.55	−0.03
Base Motor -> Back Motor	MATLAB	SolidWorks	Result	MATLAB	SolidWorks	Result	MATLAB	SolidWorks	Result
Degree	40	40		50	50		60	60	
x	−13.31	−12.96	0.35	−11.17	−10.89	0.28	−8.68	−8.5	0.18
y	11.17	10.75	0.42	13.31	12.82	0.49	15.04	14.51	0.53
z	19.55	19.46	0.09	19.55	19.47	0.08	19.55	19.48	0.07
Base Motor -> Upper Motor	MATLAB	SolidWorks	Result	MATLAB	SolidWorks	Result	MATLAB	SolidWorks	Result
Degree	40	40		50	50		60	60	
x	−4.4	−4.47	0.07	−0.85	−0.93	0.08	2.71	2.63	0.08
y	20.01	19.98	0.03	20.47	20.43	0.04	20.31	20.25	0.06
z	36.23	36.28	0.05	36.23	36.23	0	36.23	36.31	0.08
Base Motor -> Side Motor	MATLAB	SolidWorks	Result	MATLAB	SolidWorks	Result	MATLAB	SolidWorks	Result
Degree	40	40		50	50		60	60	
x	−16.23	−16.19	0.04	−12.91	−12.88	0.03	−9.19	−9.2	0.01
y	17.7	17.58	0.12	20.25	20.09	0.16	22.19	22	0.19
z	39.36	39.17	0.19	39.36	39.19	0.17	39.36	39.2	0.16

## Data Availability

The datasets generated and analyzed during the current study are available from the corresponding author upon reasonable request.
